# Development of Alkali-Activated Tiles Based on Metakaolin and Ceramic Tile Waste by Uniaxial Pressing

**DOI:** 10.3390/ma19132840

**Published:** 2026-07-03

**Authors:** Giulia Masi, Antonietta Settino, Giovanni Ridolfi, Denia Mazzini, Maria Chiara Bignozzi

**Affiliations:** 1 Department of Civil, Chemical, Environmental and Materials Engineering, University of Bologna, Via Terracini 28, 40131 Bologna, Italy; giulia.masi5@unibo.it (G.M.); antoniettasettino@gmail.com (A.S.); 2 Centro Ceramico, Via Valle d’Aosta 1, 41049 Sassuolo, Italy; ridolfi@centroceramico.it; 3 Colorobbia Italia S.p.A., Via Bucciardi 37, Fiorano Modenese, 41042 Modena, Italy; mazzinid@colorobbia.it

**Keywords:** pressing, alkali-activated materials, tiles, porosity, glazing, ceramic tile waste, thermal stability

## Abstract

**Highlights:**

- Alkali-activated tiles were prepared by uniaxial pressing.
- The water absorption and modulus of rupture requirements specified for the BIII group were satisfied.
- The investigated alkali-activated tiles exhibited thermal stability up to 850 °C suitable for glazing process.
- The compatibility of Glaze 2 with the alkali-activated substrate containing CTW was successfully validated.

**Abstract:**

This study aims to demonstrate the feasibility of transferring alkali activation technology to the ceramic tile production by developing tiles based on metakaolin and ceramic tile waste by uniaxial pressing. Optimization of the tile formulations was achieved by adjusting precursor composition (metakaolin or combination of metakaolin and ceramic tile waste), NaOH molarity, and overall water content. After uniaxial pressing at 50 MPa, alkali-activated tiles were consolidated at 50 °C for 24 h. Physical and mechanical properties were assessed following ISO test methods for ceramic tiles, allowing direct comparison with the ISO classification requirements. The more promising formulations highlight a water absorption by vacuum test method (ISO 10545-3) equal to 15%, thus allowing a BIII classification according to ISO 13006. Finally, thermal stability up to 850 °C was determined, thus allowing alkali-activated tiles to be industrially glazed.

## 1. Introduction

Amorphous aluminosilicate powders undergo chemical reactions upon contact with highly concentrated alkaline solutions [1,2]. In theory, any alkali metal can be used for alkali activation of aluminosilicates. However, most research has focused on the use of sodium and potassium sourced from Na- or K-based hydroxide and/or silicate solutions [3]. This reaction results in the formation of a 3D Si-Al-O network, typically referred to as a gel [4]. Alkali-activated materials (AAM) or geopolymers can consolidate at temperatures in the range of 20–80 °C and rapidly develop significant mechanical properties [5]. The most used precursor is metakaolin in powder form. Furthermore, various studies have highlighted the reactivity of industrial by-products, such as blast furnace slag, fly ash, rice husk, silica fume, ceramic waste, and recycled glass, which can also serve as precursors in the production of AAMs, thereby reducing their environmental impact [6,7,8,9,10,11]. Interest in these materials has grown rapidly since 2000 [12], as they were initially investigated as a sustainable alternative to cement binders and cement-based materials [13,14,15]. Optimized mix designs exhibit excellent mechanical properties, thermal insulation characteristics, fire, and chemical resistance [16,17,18].

While AAMs have been largely investigated as alternatives to traditional cementitious materials thanks to their low environmental impact and favorable properties, a limited number of studies concern their use as an alternative to traditional ceramic products, such as bricks and tiles. Although the ceramic tile industry has achieved high levels of waste management and recycling [19], particularly in Italy, it remains a sector responsible for high greenhouse gas emissions, primarily due to raw material transportation and high temperature firing (i.e., >1000 °C) [20]. Consequently, the implementation of alkali activation technology in ceramic tile manufacturing has the potential to reduce the sector’s environmental impact. Alkali-activated tiles can be produced starting from locally available natural materials and industrial by-products, exploiting consolidation temperatures much lower compared to traditional ceramics [21].

Recently, cold sintering, intended as an innovative manufacturing process which densifies ceramic materials at relatively lower temperatures (below 300 °C) than conventional hot sintering [22], has been applied to aluminosilicates (e.g., volcanic ash from Mount Etna, laterite and fly ash) using small amounts of alkaline activators [23,24,25,26]. It was demonstrated that ceramics can be prepared from volcanic ash and KOH by applying a pressure ranging from several hundred MPa up to 500 MPa, followed by heating at temperatures below 200 °C [23]. Similarly, microstructure densification has been reported for the cold sintering of laterite and rice husk ash, achieving an open porosity <10% and a density exceeding 2.0 g/cm^3^ [24]. Cold sintering was also applied for recycling geopolymer waste to promote densification of geopolymer-zeolite composites for applications in environmental remediation [26].

In previous studies [27,28,29], pressing was used to prepare alkali-activated supports with tailored porosity for asymmetric membranes in the field of wastewater treatments, particularly for oil-in-water emulsions. It was found that increasing the uniaxial pressure up to 20 MPa, the pressed support exhibits optimized characteristics such as density of 1.9 g/cm^3^ and open porosity of 16% [25]. Sheen-Ween et al. [30] also demonstrated that pressing is superior compared to casting for producing high-strength fly ash-based geopolymers with reduced alkaline solution content. Another approach reported in the literature to improve mechanical properties involves coupling high uniaxial pressure (200 MPa) with the addition of epoxy resin [31]. These inorganic-organic composites were optimized with a 4 wt% epoxy resin, promoting a dual effect of densification induced by the forming method and the resin filling gaps between particles [31].

Building on these findings, this study aims to develop alkali-activated tiles formed by pressing as a more sustainable alternative to conventional ceramic tiles. The applied methodology is fully experimental as based on previous studies [11,27,28,29,32]. In recent years, modelling and machine learning approaches have increasingly been applied to the study of geopolymers and alkali-activated materials [33,34,35]. Nevertheless, the majority of these investigations have been directed toward correlating raw material characteristics, including mix design and chemical composition, with the properties of cast products, such as workability and compressive strength in geopolymer mortars and concretes.

In this paper, the experimental approach used metakaolin (MTK) and ceramic tile waste (CTW), a by-product of the ceramic industry, with the objective of maintaining maximum compatibility with conventional ceramic tile manufacturing processes. Based on the preliminary findings reported in [32], in which NaOH molarity, water-to-precursor ratio, curing temperature, and pressure were systematically varied, this study focuses on two optimized formulations based on a binary MTK–CTW system. The tiles were produced by uniaxial pressing at 50 MPa using different amounts of NaOH solutions of 8 M and 10 M, and two different water-to-precursor ratios. The fulfilment of the main requirements for ceramic tile classification according to ISO 13006 [36], namely water absorption and modulus of rupture, together with the assessment of thermal stability and the feasibility of glaze application, is discussed to establish the first steps toward the transfer of alkali-activation technology to the ceramic tile sector.

## 2. Materials and Methods

### 2.1. Materials

Two different precursors were selected for the preparation of alkali-activated tiles: a flash calcined MTK and CTW, a by-product sourced by the rectifying process used to trim and dimension porcelain stoneware tiles. In detail, MTK (Argicem), kindly sourced from Argeco Développement (Fumel, France), was produced by a flash calcination process at 700–750 °C for a few tenths of a second and then rapidly quenched to 100 °C [37,38]. CTW was kindly sourced from Ascot Everytile (Sassuolo, Modena, Italy). The chemical composition of MTK and CTW is reported in Table 1 and was determined by Inductively Coupled Plasma-Optical Emission Spectroscopy (Avio 550 MAX ICP-OES, PerkinElmer, Milan, Italy). Both precursors exhibit a similar chemical composition, with silicon and aluminum oxides accounting for more than 90 wt% of the total composition. Nevertheless, the amorphous content of MTK and CTW differs significantly, being 29% and 50%, respectively, as determined elsewhere [11,39,40,41,42]. These values were used to calculate the theoretical molar ratios during the mix design stage [5].

Table 2 reports the particle size distribution characteristic values (d(10), d(50), and d(90)), specific surface area (SSA), and specific pore volume (SPV) for MTK and CTW. These physical characteristics were determined by a Malvern Mastersizer 2000 particle analyzer (Lissone, Monza, Italy), and a N_2_ adsorption surface area analyzer applying the Brunauer–Emmett–Teller (BET) theory (Anton Paar NOVA 800, Rivoli, Turin, Italy). Despite MTK showing a higher particle size compared to CTW, it exhibits higher SSA and SPV values than CTW. This is due to an agglomeration effect of MTK particles produced by flash calcination already observed elsewhere [11,39,42], which leads to a coarser particle size distribution, but significantly higher overall porosity.

The activating solution was prepared by mixing sodium hydroxide and sodium silicate solutions. The latter, with the commercial name of Reoflux B, was kindly supplied by Ingessil srl, Verona, Italy. The nominal composition of the Na_2_SiO_3_ solution is SiO_2_ = 29.9 wt%, Na_2_O = 14.4% and H_2_O = 55.7 wt% and SiO_2_/Na_2_O ratio equal to 2.1. NaOH solutions (8 and 10 M) were prepared starting from NaOH pellets (ACS reagent, Sigma Aldrich, Milan, Italy) at least 24 h before mixing and were left at room temperature to reach the chemical equilibrium.

### 2.2. Sample Preparation

A preliminary study [32] investigated several alkali-activated formulations based on metakaolin to evaluate the best operative conditions in terms of pressing load, curing temperature and NaOH molarity. Based on previous findings reported in refs. [27,28,29,32], five formulations were prepared by combining metakaolin (MTK) and ceramic tile waste (CTW) and using different amounts of NaOH solutions at two concentrations (8 M and 10 M). The different formulations resulted in water-to-precursor ratios (w/p) of 0.14 and 0.21, as summarized in Table 3. Samples designated as PMK and PCW were prepared using only MTK or a blend of MTK and CTW, respectively. A constant MTK replacement of 20% with CTW was adopted. The obtained tiles were named following this nomenclature: PMK or PCW stands for pressed tiles obtained from 100% MTK or from 80% MTK + 20% CTW, respectively. Then, the concentration of NaOH solution is reported followed by the total water content. Thus, PMK-8M-15 indicates a mix with MTK as precursor, 8 M NaOH and 15 wt% of total water. Table 3 also reports the theoretical molar ratios in terms of amorphous SiO_2_/Al_2_O_3_, Na_2_O/Al_2_O_3_ and Na_2_O/amorphous SiO_2_. The SiO_2_/Al_2_O_3_ ratio slightly differs between mixes prepared with only MTK or a blend of MTK and CTW. The other two molar ratios, i.e., Na_2_O/Al_2_O_3_ and Na_2_O/amorphous SiO_2_, vary depending on the concentration of NaOH solution and the total water amount, respectively.

**Figure 1:**
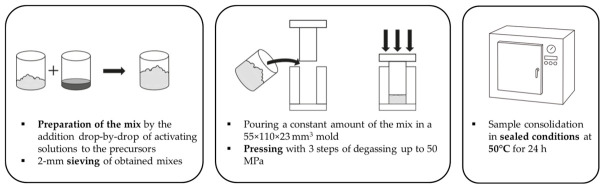
Stages of the alkali-activated tile preparation process by pressing.

Alkali-activated tiles were prepared according to the scheme reported in Figure 1. In detail, precursors, i.e., MTK or a blend of MTK and CTW, were homogenized by stirring in a plastic container, and the activating solution, i.e., a blend of NaOH and Na_2_SiO_3_ solutions, was added drop-by-drop, in order to have a homogenous distribution. The time required for the addition of the activating solutions was 3 min. An additional minute was used to continuously stir the mixture. Then, it was sieved using a 2 mm sieve in order to obtain a homogenous mix characterized by fine particles. The total preparation time was 5 min. Rectangular prismatic specimens (55 × 110 × 23 mm^3^) were prepared by filling steel molds with the mix and compacting them using a uniaxial press (Minipressa, SACMI, Imola, Italy) at a maximum pressure of 50 MPa for 3 s. To optimize the forming procedure and facilitate air removal, three degassing steps were applied during pressing at 7.5, 15, and 22 MPa before reaching the final pressure of 50 MPa. At least 10 samples were prepared for each formulation. After pressing, tiles were wrapped in a plastic bag and heated at 50 °C for 24 h. Then, after demolding, tiles were stored, always wrapped in the plastic bag, at room temperature for an additional 6 days before testing. The curing conditions were selected on the basis of previous findings [32,40].

### 2.3. Characterization

The whole characterization of alkali-activated tiles obtained by pressing was carried out after 7 days from their preparation. To determine the geometric density, the water absorption and the stability in water by assessment of the mass loss, samples were first dried in an oven at 100 °C for at least 24 h to reach a constant dry mass (m_d_). The geometric density was calculated as the ratio of the dry mass (m_d_) and the geometric volume of the samples.

Water absorption (WA) was determined by the under-vacuum test method according to ISO 10545-3:2018 [43]. After evacuating the samples to a pressure of around 10 KPa, the samples were submerged in deionized water while the vacuum was maintained for at least 10 min. It was waited to reach sample saturation, and then samples were weighed in saturated-surface-dry condition (m_ssd_). Water absorption was calculated in accordance with the following equation (Equation (1)):

WA, % = 100 · [(m_ssd_ − m_d_)/m_d_] (1)

After immersion in water for 24 h, samples were dried again in an oven at 100 °C for at least 24 h and the dry mass (m_d2_) was determined to measure the mass loss as an indication of the stability of samples in water, in accordance with the following equation (Equation (2)):

Stability in water, % = 100 · [(m_d_ − m_d2_)/m_d_] (2)

The reported results for geometric density, water absorption and water stability are an average of at least six measurements.

The breaking load and the modulus of rupture were determined by a three-point bending test using a strength tester (CC 96/2006, Nannetti s.r.l., Faenza, Italy) in accordance with ISO 10545-4:2019 [44]. Two oscillating supports that held the sample were positioned with a span of 90 mm. The loading was carried out by moving the upper blade at a rate of 1 MPa/s. The breaking load in Newtons (N) was recorded and the modulus of rupture was calculated in accordance with the following equation (Equation (3)):

Modulus of Rupture, MPa = (3F_max_ · l)/(B · h^2^), (3)

where F_max_ is the breaking load (in N), l is the span between the supports (in mm), B is the short edge of the sample (in mm), and h is the minimum thickness of the sample measured after the test along the broken edge (in mm). The reported results are an average of 10 measurements.

Open porosity and pore size distribution were investigated for all five formulations by means of mercury intrusion porosimetry (MIP). The equipment (Thermo Fisher Scientific, Tecmat s.r.l., Como, Italy) consists of two units: Pascal 140 unit measures the open porosity in the range of 3.8–116 µm by varying the pressure between 100 and 400 kPa, while Pascal 240 unit measures the open porosity in the range of 0.0074–15 µm by varying the pressure in the range of 0.1–200 MPa. The Washburn equation (Equation (4)) was applied to express the pore size as a function of the applied pressure, considering a capillary flow in cylindrical pores, as follows [45]:

P = (2 γ cos θ)/r(4)

where P is the applied pressure, γ is the Hg surface tension equal to 0.48 N/m, θ is the Hg contact angle equal to 141.3° and r is the pore radius. S.O.L.I.D. software (SOLver of Intrusion Data, Ver. 1.6.2 (29 September 2015) Thermo Scientific, Waltham, MA, USA) was used to manage the obtained data.

The morphology of the consolidated alkali-activated tiles was observed by a Field Emission Gun-Scanning Electron Microscope (FEG-SEM), applying a Mira3 FEG-SEM (Tescan Group a.s., Brno, Czech Republic) by observing free surfaces. Images were recorded using secondary electrons by applying a voltage of 15 kV and a working distance of 10 mm. Before SEM observation, samples were sputter-coated with gold to make them conductive using a Q150R ES sputter coater (Quorum Technologies Ltd., West Sussex, UK).

The evaluation of the 3D network formation by alkali activation was assessed by Fourier Transform-Infrared Spectroscopy (FT-IR) using a Spectrum Two instrument (PerkinElmer, Milan, Italy) in ATR mode in the range of 2000–400 cm^−1^. The applied spectral resolution was 4 cm^−1^, and 16 scans were acquired with a data interval of 1 cm^−1^.

A hot stage microscope (HSM) was used to test the thermal volumetric stability of each formulation by preparing small cubic samples with an edge of about 3 mm. The procedure consists of heating each sample in an oxidizing atmosphere, and in parallel, images of its silhouette are continuously collected. The initial sample height is recorded, and its percentage is considered 100% at the beginning of the analysis. The test was carried out using the Misura^®^ HSM 3.32 heating microscope (Expert System Solutions, Modena, Italy) with a heating rate of 5 °C/min from ambient temperature to 1315 °C in air.

Lastly, preliminary glazing tests were carried out on the investigated alkali-activated tiles by applying commercial glazes kindly supplied by Colorobbia Italia S.p.A (Fiorano Modenese, Modena, Italy). Two different commercial lead-free glazes were applied on a small portion of the pressed tiles (30 × 55 mm^2^) with the following features:

- Glaze 1: transparent glaze applied with a density of 1550 g/L. Firing temperature: 820 °C for 60 min;
- Glaze 2: transparent glaze with carboxymethyl cellulose, with a density of 1550 g/L. Firing temperature: 840 °C for 50 min.

Before glaze application, all the samples were decorated with a color pattern using a digital printer (Digiglaze, System Ceramics S.p.A, Fiorano Modenese, Modena, Italy). Visual inspection was performed on the quality of the applied glaze by photographic documentation.

## 3. Results and Discussion

### 3.1. Physical and Mechanical Properties and Microstructure

**Figure 2:**
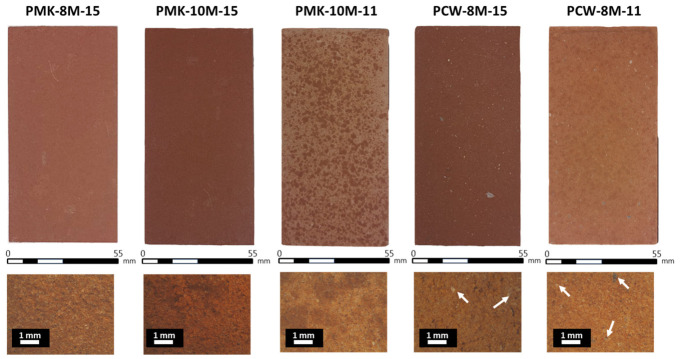
Macros (**top**) and optical microscopy (**bottom**) images of the alkali-activated tile surfaces. White arrows highlight the presence of unreacted CTW particles.

By applying the procedure described in Section 2.2, five sets of alkali-activated tiles were produced. Although all the tested formulations were successfully formed by pressing, qualitative differences in their behavior were observed through visual inspection of the tile surfaces. A representative surface for each formulation is reported in Figure 2. Some considerations are listed as follows:

- Regardless of the type of precursor and the NaOH molarity, the total water content of 15 wt% (i.e., PMK-8M-15, PMK-10M-15 and PCW-8M-15) leads to a more homogenous surface than a reduced content of 11 wt% used for the preparation of PMK-10M-11 and PCW-8M-11. The latter tiles exhibited several surface heterogeneities, characterized by small areas with different colors. This observation suggests that the amount of total water plays a crucial role in ensuring the homogeneous distribution of the activating solution throughout the mixture.
- When MTK is partially replaced with CTW (i.e., PCW-8M-15 and PCW-8M-11), unreacted CTW particles are observed on tile surfaces. The presence of these particles is more evident in the optical microscopy images, in which they are highlighted by white arrows (Figure 2, bottom row). Their presence suggests that the current CTW grain size distribution includes too many coarse particles that are poorly reactive in the investigated conditions. A preliminary milling treatment of CTW would likely enhance its reactivity in alkaline environments by increasing its specific surface area and, consequently, promoting dissolution processes.

The above-mentioned observations indicate that the balance between precursor fineness and water content must be carefully optimized to ensure the successful application of alkali activation technology in tile production.

**Figure 3:**
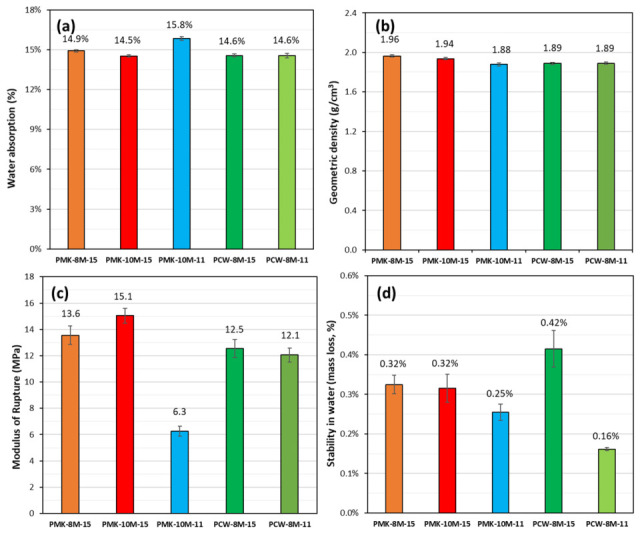
Physical and mechanical properties of the investigated alkali-activated tiles: results of (**a**) under vacuum water absorption test, (**b**) geometric density, (**c**) modulus of rupture, and (**d**) mass loss after water immersion for 24 h.

The qualitative differences identified through the observation of the pressed tile surfaces are consistent with the physical and mechanical properties summarized in Figure 3. Starting from the results related to the under-vacuum water absorption test (Figure 3a), which is conventionally used to classify ceramic tile products according to ISO 13006:2018 [36], all five sets of pressed tiles exhibited water absorption values in the range 14.5–14.9%, except for PMK-10M-11, which exhibited a slightly higher value around 15.8%. According to the classification reported in ISO 13006, all the tested formulations are compliant with the BIII group. This group includes wall ceramic tiles typology, normally characterized by water absorption values greater than 10%. The geometric density values (Figure 3b) are in a range between 1.88 and 1.96 g/cm^3^. PMK-10M-11 exhibits the lowest density, and a value of 1.89 g/cm^3^ was found for the samples prepared with the blend of MTK and CTW. In previous studies [11,40,42], CTW was applied as a precursor for alkali activation in combination with fly ash or flash calcined metakaolin to prepare lightweight composites or sustainable binders, respectively. In these studies, a decreasing trend in geometric density was determined when CTW content increased, indicating a lower reactivity of CTW compared to fly ash or flash calcined metakaolin.

The modulus of rupture values reported in Figure 3c exhibited an inverse trend compared to water absorption results, as expected. PMK tiles with only MTK as precursor and a total water content of 15% show the highest values, reaching up to 15.1 MPa. On the contrary, PMK-10M-11 exhibits a very modest modulus of rupture that is less than half compared to that determined with the same mix but with 15 wt% of total water content. The combined use of MTK and CTW as precursors in PCW samples leads to tiles with a modulus of rupture close to 12.5 MPa, confirming the slightly lower performance because of the introduction of CTW as a precursor. Nevertheless, except for formulation PMK-10M-11, all mixes satisfy the modulus of rupture requirement specified by ISO 13006:2018 for the BIII group. Moreover, the obtained values are in good agreement with those reported in previous studies on alkali-activated materials based on different precursors, where similar uniaxial pressing pressures and curing temperatures below 100 °C were employed [24,27].

As an index of the stability of the material and alkali activation degree [40,46], the mass loss of each tile was recorded after 24 h of water immersion (Figure 3d). All the samples remained intact after testing, and the maximum mass loss of 0.42 wt% was recorded for PCW-8M-15, likely indicating that only a few unreacted alkaline salts were leached out during water immersion. Accordingly, pressed tiles with a reduced content of alkaline solutions exhibit the lowest mass loss, i.e., 0.25 wt% for PMK-10M-11 and 0.16 wt% for PCW-8M-11.

**Figure 4:**
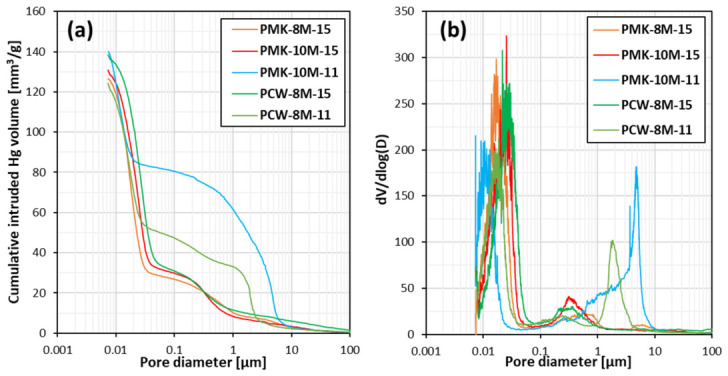
Pore size distribution by MIP: (**a**) cumulative and (**b**) derivative distributions of open pores.

As physical and mechanical properties of porous materials are also influenced by their open porosity, this one was measured by MIP, and the results are reported in Figure 4 as cumulative and derivative curves based on the measurement of the intruded Hg. All the pressed tiles show a similar level of total open porosity around 20–25%, corresponding to a cumulative intruded Hg volumes of 120–140 mm^3^/g (Figure 4a). However, the pore size distribution is different for samples containing 15 wt% of total water content compared to those containing 11 wt%. Indeed, both PMK-10M-11 and PCW-8M-11 are characterized by a pronounced bimodal pore size distribution, with peaks located at approximately 0.01 and 0.03 µm. Furthermore, a substantial porosity contribution is observed in the 1–4 µm range for PCW-8M-11 and in the 0.1–10 µm range for PMK-10M-11 (Figure 4b). Wang et al. [31] have observed the same bimodal behavior when a blend of MTK and fly ash was alkali-activated and then pressed using a uniaxial pressure of 200 MPa. On the contrary, in previous studies [27,28] a unimodal pore size distribution was measured with a typical pore diameter of 1.0 µm, when metakaolin was activated with anhydrous sodium silicate and pressed with a maximum pressure of 20 MPa. Therefore, the influence of the applied uniaxial pressure on the pore size distribution is evident. In particular, uniaxial pressures equal to or greater than 50 MPa appear to promote the formation of a bimodal pore size distribution, including a significant fraction of nanopores in the range of 10–30 nm.

**Figure 5:**
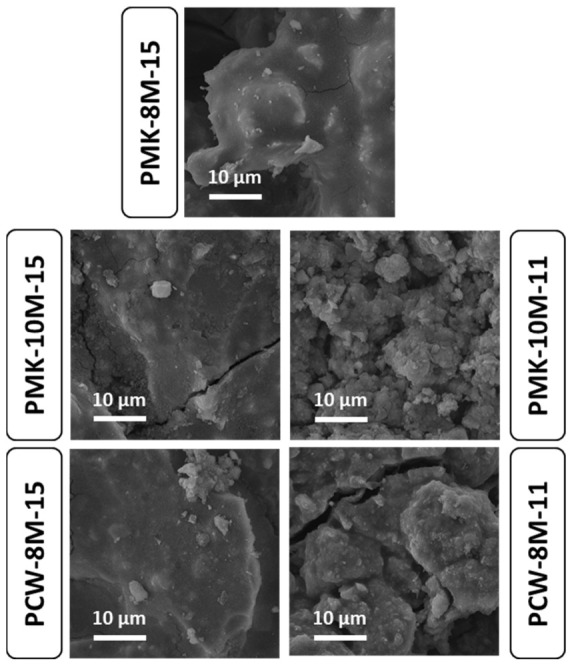
SEM images of the surfaces of alkali-activated tiles at two different magnifications.

SEM observation of the free surfaces of alkali-activated tiles was carried out to observe their microstructure. Micrographs are reported in Figure 5: dense and continuous microstructures are detected for samples with a water content of 15 wt%, while PMK-10M-11 and PCW-8M-11 exhibit a discontinuous and a powder-like shaped microstructure in agreement with the high amount of large open pores detected by MIP. Some unreacted particles are also observed in these samples. These can be ascribed to the quartz contained in the flash calcined metakaolin [28], which cannot actively participate in alkali activation, or to unreacted CTW particles.

**Figure 6:**
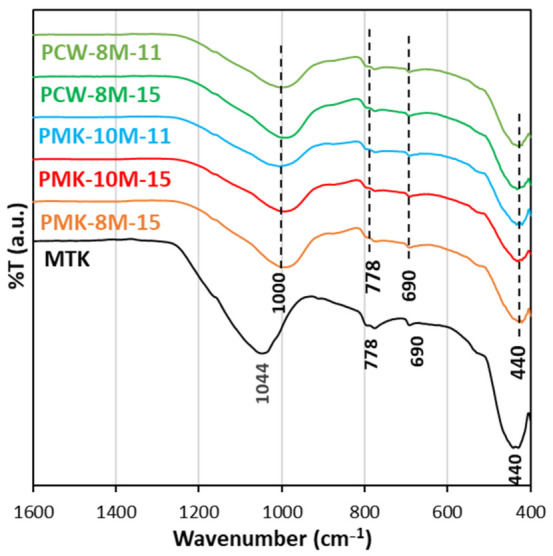
FT-IR spectra of the alkali-activated tiles and MTK.

Lastly, to confirm that the 3D network typical of alkali activation was formed, the FT-IR spectra recorded for all five tile formulations are reported in Figure 6. For comparison’s sake, FT-IR of MTK is also reported. In MTK, contributions related to Si–O–Si stretching vibration (1044 cm^−1^), Al–O stretching vibration (778 cm^−1^), vibration of Al–O–Si (690 cm^−1^) and bending of Si–O–Si (440 cm^−1^) were detected [47,48]. The FT-IR spectra of the alkali-activated tiles differ from that of MTK mainly by the shift of the Si–O–Si stretching band from about 1044 cm^−1^ to 1000 cm^−1^. This shift, associated with Si–O–Al stretching vibrations, confirms the formation of the three-dimensional aluminosilicate network characteristic of alkali activation [48].

### 3.2. High Temperature Behavior and Glazing

One of the most significant properties of alkali-activated materials (AAMs) is their excellent high-temperature resistance, a key characteristic that can be particularly beneficial in several industrial sectors and civil engineering applications [49].

The current technology applied in the ceramic tile industry involves single or double firing at temperatures in the range of 800–1250 °C, where support and glaze can be fired together (single firing) or in two different processes (double firing).

**Figure 7:**
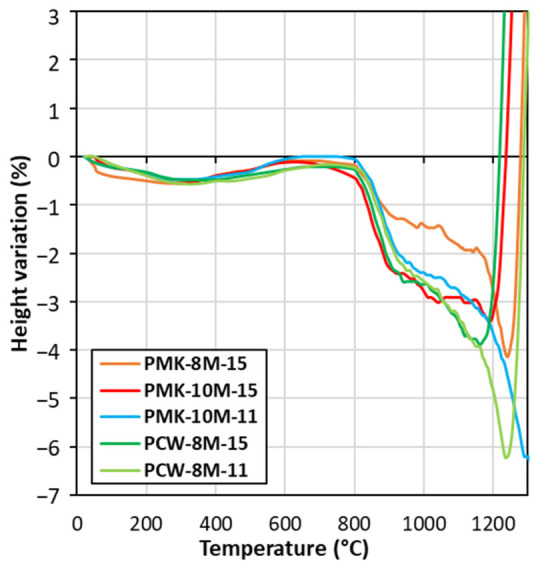
Variation of the height (%) of the investigated alkali-activated during hot stage microscope testing.

One of the objectives was therefore to verify whether low-melting-temperature glazes (T_m_ = 800–850 °C), commonly used in double-firing ceramic processes, are suitable for application on alkali-activated tiles and whether the latter remain thermally stable within this temperature range. For this reason, results in terms of sample height variation obtained by HSM—a typical test method used in ceramic tile research and development [50]—are reported in Figure 7 and values of height reduction at characteristic temperatures are reported in Table 4. Until 800 °C, comparable behavior was observed for all five tested tiles, with a small variation of the sample silhouette lower than 0.5% as height reduction. This small change can be ascribed to the dehydration of free or weakly bound water between 100 and 200 °C and certain structural reorganization between 300 and 600 °C, as reported in literature [51,52].

**Figure 8:**
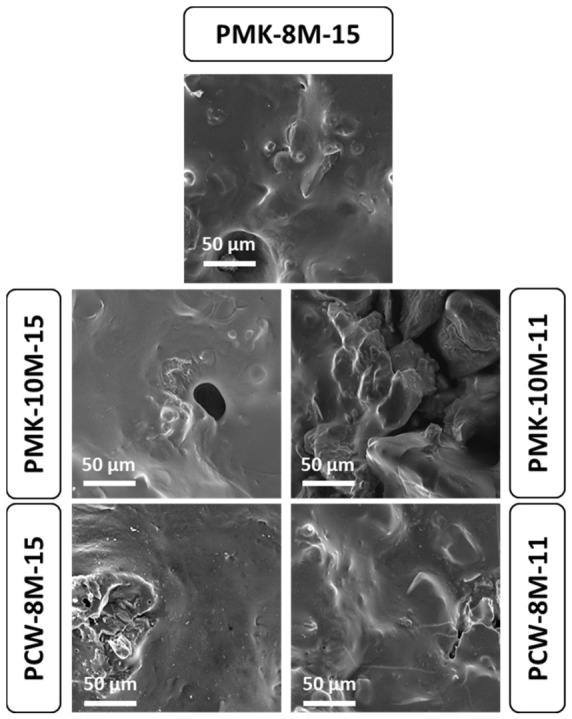
SEM images of surfaces of alkali-activated tiles at the end of the hot stage microscope test.

Significant changes occur for temperatures higher than 850 °C, with a contraction of the samples greater than 1% after 900 °C. Samples continued to shrink by increasing temperature, with the highest shrinkages located in the range of 1150–1300 °C, probably due to densification processes related to sintering [53]. Evidence of the densification of the tiles after the HSM test has also been observed by SEM on the tile surface (Figure 8). All the samples exhibit a continuous and vitreous surface compared to the observation carried out after consolidation (Figure 5). Finally, after the highest shrinkage values, a pronounced expansion was observed in the HSM measurements for all samples, likely associated with recrystallization phenomena occurring at very high temperatures [54].

HSM analysis allowed the thermal stability of all five alkali-activated tile formulations to be verified up to 850 °C, thereby enabling the subsequent decoration and glazing procedures, which constituted one of the main objectives of this study. A more comprehensive understanding of the high-temperature behavior of alkali-activated tiles, including phase evolution and transformation mechanisms, would require additional investigations such as thermogravimetric and differential scanning calorimetry analyses (TGA/DSC) and high-temperature X-ray diffraction (HT-XRD). However, these analyses were not included in the present work, as they were considered beyond the scope of the study and not essential for evaluating the suitability of the investigated tiles for low-temperature glazing processes.

**Figure 9:**
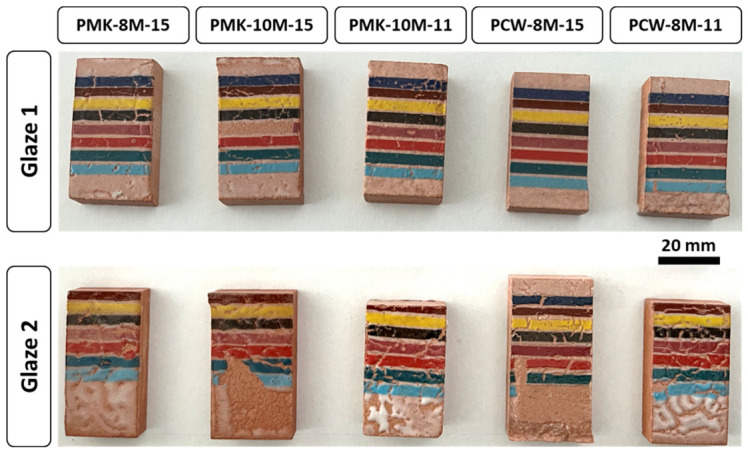
Images of decorated and glazed alkali-activated tiles.

The application of low-melting commercial lead-free glazes and the relevant firing process was carried out in an industrial context to mimic a real industrial process potentially suitable for alkali-activated tiles. Results of the decoration and glazing process is shown in Figure 9, where visual observation of the glazed surface appearance is reported.

Glaze 1 exhibits some small defects, such as crazing and pinholes, when applied to PMK-based samples. Defects are less evident on PCW-8M-11 and fully disappear on PCW-8M-15, thus indicating better compatibility when ceramic waste is also present in the alkali-activated mixes. Glaze 2 exhibits evident swellings and detachments on all the different supports, indicating its incompatibility. Although preliminary results for Glaze 1 are promising, the development of specifically designed glaze formulations is needed to avoid the formation of defects on metakaolin-based geopolymers. Moreover, the possibility of applying glazes which require lower melting temperatures is particularly attractive for future investigations, as this aspect can further improve the overall environmental sustainability of alkali-activated tiles.

## 4. Conclusions

This study aimed to develop alkali-activated tiles based on flash-calcined metakaolin and ceramic tile waste, formed via uniaxial pressure. The main conclusions are as follows:

- Five different formulations were successfully designed for the preparation of alkali-activated tiles. Reduced water content resulted in heterogeneity and modest physical and mechanical properties, due to difficulties in homogenously distributing the alkaline activators throughout the sample. However, all tiles prepared with 15 wt% water, regardless of the precursors or NaOH concentration, exhibit water absorption values comparable to commercial wall tiles belonging to the BIII group (ISO 13006:2018). Further studies are currently underway to optimize the formulations, reduce water absorption, and ultimately achieve the performance levels required for tiles classified within the BII and BI groups according to ISO 13006;
- Uniaxial pressure of 50 MPa induced a bimodal pore-size distribution, with the primary population in the nanoporosity range (10–30 nm). The amount of alkaline activating solution influenced the formation of a secondary pore population. Reducing the alkaline solution content resulted in a coarsening of the pore size distribution by one order of magnitude compared to tiles prepared with 15 wt% water, which impaired the modulus of rupture;
- All alkali-activated tiles exhibited high thermal stability, making them promising candidates for decoration and glazing procedures conventionally used in ceramic tile production. Minimal shrinkage was measured in all samples up to 800 °C. At 1200 °C, samples prepared with an 8M NaOH solution displayed the lowest shrinkage, highlighting their potential also for high-temperature applications.
- Preliminary results using Glaze 1, a commercial lead-free glaze, were encouraging. The presence of ceramic tile waste in the support appears to improve the compatibility with the glaze. Overall, future research should focus on the development of low-temperature glaze formulations specifically designed for alkali-activated materials, with the aim of further enhancing their environmental sustainability.

Finally, this study constitutes a significant initial step toward the technological transfer of alkali activation to the ceramic tile industry. However, additional research is needed to evaluate long-term durability, service-life performance, and the potential for industrial-scale production of alkali-activated tiles.

## Figures and Tables

**Table 1:** Chemical composition (wt%) of precursors, i.e., MTK and CTW, determined by ICP-OES. LOI stands for Loss of Ignition, and LoD stands for Limit of Detection.

	SiO_2_	Al_2_O_3_	TiO_2_	Na_2_O	K_2_O	CaO	MgO	Fe_2_O_3_	ZrO_2_	SO_3_	Cr_2_O_3_	BaO	LOI
MTK	72.0	22.1	1.0	0.04	0.3	0.4	0.1	1.6	<LoD	<LoD	0.02	<0.01	2.4
CTW	73.2	17.5	0.8	2.9	1.9	1.5	0.5	1.1	0.4	0.1	<LoD	<LoD	0.4

**Table 2:** Physical properties, i.e., particle size distribution characteristic values (d(10), d(50) and d(90)), Specific Surface Area (SSA) and Specific Pore Volume (SPV) of MTK and CTW.

	d(10) μm	d(50) μm	d(90) μm	SSA (m^2^/g)	SPV (cm^3^/g)
MTK	6.5	42.0	121.1	11.0 ± 0.7	0.064 ± 0.003
CTW	3.3	23.3	91.4	0.68 ± 0.01	0.0035 ± 0.0002

**Table 3:** Formulations (wt%) for preparing pressed tiles by ranging NaOH molarity and total water content. Theoretical molar ratios and water-to-precursor (w/p) ratios are also reported.

Sample Name	MTK	CTW	8M NaOH Solution	10M NaOH Solution	Na_2_SiO_3_ Solution	SiO_2_/Al_2_O_3_	Na_2_O/SiO_2_	Na_2_O/Al_2_O_3_	Total H_2_O ^(a)^	w/p
PMK-8M-15	74.0	-	7.0	-	19.0	2.8	0.15	0.50	15	0.21
PMK-10M-15	74.0	-	-	7.0	19.0	2.8	0.20	0.50	15	0.21
PMK-10M-11	81.0	-	-	5.0	14.0	2.8	0.15	0.35	11	0.14
PCW-8M-15	58.0	15.0	6.0	-	21.0	3.2	0.20	0.50	15	0.21
PCW-8M-11	64.0	17.0	4.0	-	15.0	3.2	0.15	0.35	11	0.14

^(a)^ Total H_2_O is the water (in %) used for the preparation of NaOH and Na_2_SiO_3_ solutions.

**Table 4:** Sample height reduction during hot stage microscope testing (HSM) recorded at characteristic temperatures.

Temperature (°C)	Sample Height Reduction				
	PMK-8M-15	PMK-10M-15	PMK-10M-11	PCW-8M-15	PCW-8M-11
200	0.41	0.27	0.36	0.32	0.39
400	0.41	0.49	0.42	0.49	0.50
600	0.09	0.16	0.05	0.27	0.28
800	0.18	0.27	0.05	0.27	0.22
900	1.20	2.19	1.56	2.16	1.74
1000	1.43	2.68	2.40	2.64	2.58
1100	1.84	2.90	2.76	3.40	3.31
1200	2.81	3.34	3.65	2.70	4.82

## Data Availability

The original contributions presented in this study are included in the article. Further inquiries can be directed to the corresponding author.
